# Multi-omics analysis of druggable genes to facilitate Alzheimer's disease therapy: A multi-cohort machine learning study

**DOI:** 10.1016/j.tjpad.2025.100128

**Published:** 2025-03-11

**Authors:** Jichang Hu, Yong Luo, Xiaochuan Wang

**Affiliations:** Department of Pathophysiology School of Basic Medicine Key Laboratory of Education Ministry/Hubei Province of China for Neurological Disorders Tongji Medical College, Huazhong University of Science and Technology, Wuhan, China

**Keywords:** Alzheimer's disease, Machine learning, WGCNA, Mendelian randomization

## Abstract

**Background:**

The swift rise in the prevalence of Alzheimer's disease (AD) alongside its significant societal and economic impact has created a pressing demand for effective interventions and treatments. However, there are no available treatments that can modify the progression of the disease.

**Methods:**

Eight AD brain tissues datasets and three blood datasets were obtained. Consensus clustering was utilized as a method to discern the various subtypes of AD. Then, module genes were screened using weighted correlation network analysis (WGCNA). Furthermore, screening hub genes was conducted through machine-learning analyses. Finally, A comprehensive analysis using a systematic approach to druggable genome-wide Mendelian randomization (MR) was conducted.

**Results:**

Two AD subclasses were identified, namely cluster.A and cluster.B. The levels of gamma secretase activity, beta secretase activity, and amyloid-beta 42 were found to be significantly elevated in patients classified within cluster A when compared to those in cluster B. Furthermore, by utilizing the differentially expressed genes shared among these clusters, along with identifying druggable genes and applying WGCNA to these subtypes, we were able to develop a scoring system referred to as DG.score. This scoring system has demonstrated remarkable predictive capability for AD when evaluated against multiple datasets. Besides, A total of 30 distinct genes that may serve as potential drug targets for AD were identified across at least one of the datasets investigated, whether derived from brain samples or blood analyses. Among the identified genes, three specific candidates that are considered druggable (*LIMK2, MAPK8*, and *NDUFV2*) demonstrated significant expression levels in both blood and brain tissues. Furthermore, our research also revealed a potential association between the levels of *LIMK2* and concentrations of CSF Aβ (OR 1.526 (1.155–2.018)), CSF p-tau (OR 1.106 (1.024–01.196)), and hippocampal size (OR 0.831 (0.702–0.948)).

**Conclusions:**

This study provides a notable advancement to the existing literature by offering genetic evidence that underscores the potential therapeutic advantages of focusing on the druggable gene *LIMK2* in the treatment of AD. This insight not only contributes to our understanding of AD but also guides future drug discovery efforts.

## 1 Introduction

Alzheimer's disease (AD) is a chronic and progressive neurodegenerative disorder characterized by the accumulation of amyloid-β plaques and neurofibrillary tangles in the brain, leading to a gradual decline in cognitive function and the development of dementia [1,2]. As the predominant form of dementia, AD accounts for approximately 60–80 % of dementia cases globally, imposing a considerable burden on patients, caregivers, and society [3]. With the global population aging rapidly, the prevalence of AD is projected to increase significantly, potentially doubling the global patient population by 2050 [4]. The swift rise in the occurrence of AD, coupled with its social and economic implications, has created an immediate demand for effective therapeutic approaches for this ailment.

However, treatment with AD drugs may not completely eliminate the disease [5,6]. By utilizing the insights gained from these extensive genetic analyses, researchers can identify potential therapeutic targets that are more likely to yield effective treatments, ultimately enhancing the efficiency and success rates of drug development efforts [7,8]. In essence, genes that are considered 'druggable,' which encode proteins or regulate gene expression, can offer significant insights into potential drug targets [9]. In recent years, a multitude of extensive GWASs (Genome wide association studies) have revealed a variety of SNPs (Single nucleotide polymorphism) that are linked to the risk of AD [10]. Nonetheless, one of the limitations of GWAS is that they do not explicitly and directly pinpoint causal genes nor specific drug targets. This is primarily because many of the SNPs that have been identified are situated within non-coding regions or between genes, making it challenging to establish a clear connection between these genetic variations and their functional implications in relation to drug discovery [11,12]. Mendelian randomization (MR) serves as a valuable methodological approach for evaluating the causal relationships between modifiable exposures or risk factors and clinically significant outcomes [13,14]. This technique leverages genetic variations as instrumental variables to draw inferences about causality, thus minimizing biases often associated with observational studies. By employing MR, researchers can more reliably ascertain whether changes in a specific risk factor are implicated in alterations in health outcomes, ultimately enhancing our understanding of the underlying mechanisms at play.

In light of the pressing need for effective therapeutic targets for AD, we conducted a systematic druggable genome-wide MR study. First, consensus clustering was utilized as a method to discern the various subtypes of AD, and DEGs (Differentially expressed genes) between the two subtypes were identified. Then, key module genes were identified by weighted correlation network analysis (WGCNA) between the two subtypes. Moreover, utilizing the shared cluster DEGs, key module genes, and druggable genes, 21 hub genes were obtained. Next, we filtered the hubs through multiple machine learning methods. Finally, a two-sample Mendelian randomization (MR) analysis was conducted, incorporating the identified druggable genes, to assess the causal relationships between druggable gene expression and the risk of AD. This approach aimed to provide insights into how specific genetic variants that influence gene expression could potentially impact the etiology of AD. By utilizing this methodology, the study seeks to deepen our understanding of the mechanisms that underlie AD and identify potential targets for therapeutic intervention.

## 2 Materials and methods

### 2.1 Data acquisition

**Fig. 1:**
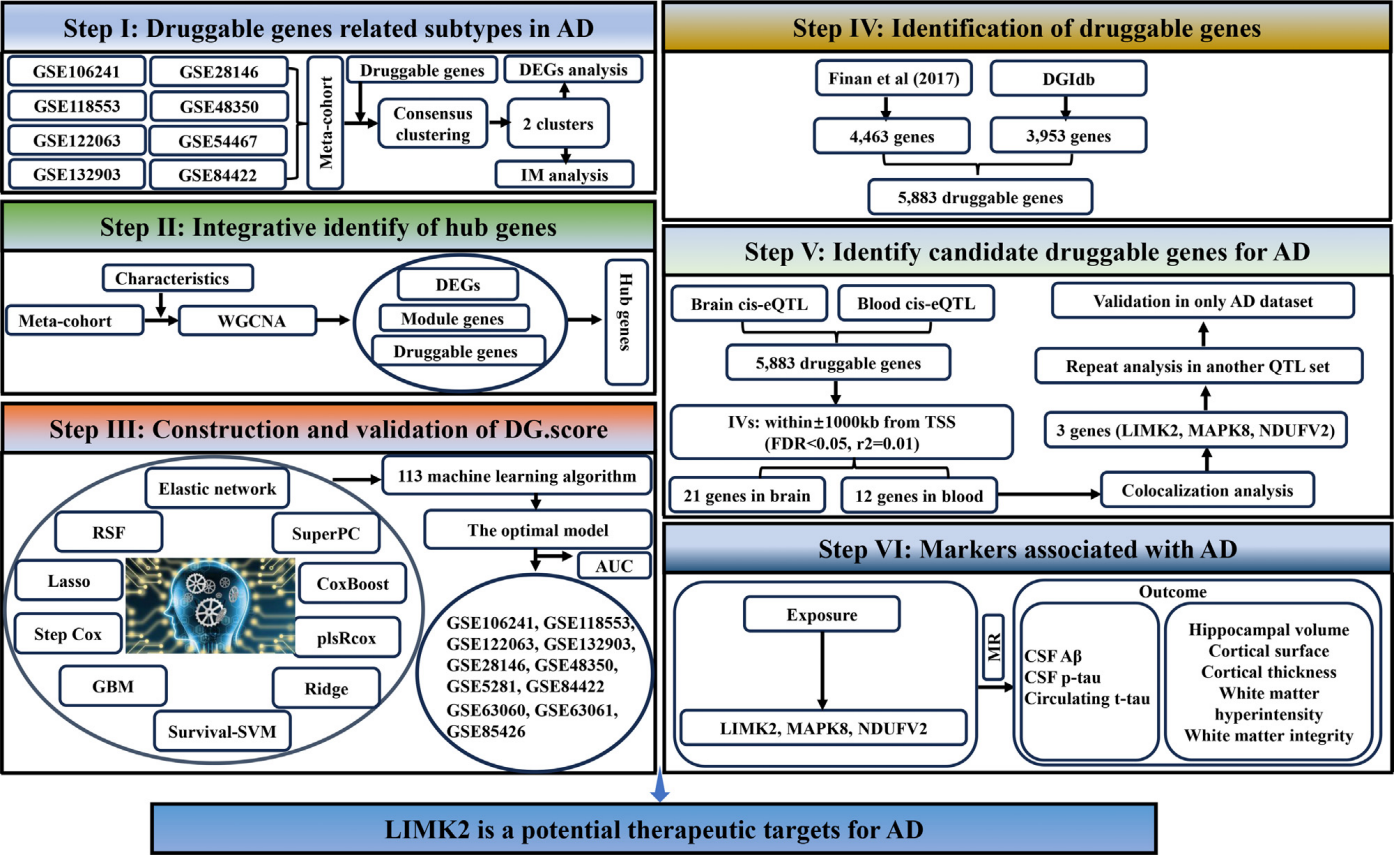
The flowchart of the whole study.

In this study, the present study systematically retrieved the microarray datasets based on the following terms: “Alzheimer's disease” or “AD” and 'Homo sapiens'. Datasets were acquired based on the following eligibility criteria: i) Samples from AD brain tissues or AD blood samples; and ii) raw data or gene expression by array were accessible in GEO (Gene Expression Omnibus). Finally, transcriptome data from the 8 CE brain tissues datasets (GSE106241 [15], GSE28146 [16], GSE118553 [17], GSE48350 [18], GSE122063 [19], GSE5281 [20], GSE132903 [21], and GSE84422 [22]) and 3 CE blood samples datasets (GSE63060 [23], GSE63061 [23], and GSE85426) were obtained. Normalization of gene expression in the four datasets and conversion of probe IDs to gene names in the datasets. To address batch effects between different databases, the datasets using the "sva" in to a meta cohort [24]. The flowchart of the entire investigation is detailed in Fig. 1.

### 2.2 Consensus unsupervised clustering analysis

In this work, consensus unsupervised clustering analysis with k-values ranging from 2 to 9 using the R package "ConsensusClusterPlus" [25]. Next, we compared DEGs in different AD subtypes.

### 2.3 DEGs and functional annotation

DEGs screening between two subtypes were identified. The mean expression value of each gene was calculated in the two groups, and the fold change value was calculated. The FDR (false discovery rate) correction algorithm was applied to correct the p-values, and DEGs were identified with adj.P.Val 〈 0.05 and |log2FC| 〉 0.585 as the significance threshold. To explore the impact of these genes on the development of AD.

### 2.4 WGCNA

The R package “WGCNA” was used to build a co-expression network aimed at identifying the gene module most closely associated with AD [26]. Initially, we determined Pearson's correlation matrices for each gene pair, leveraging the average linkage method along with a weighted correlation coefficient to form a weighted adjacency matrix. Subsequently, using a soft power parameter (β), we computed the adjacency and transformed it into a topological overlap matrix (TOM). By applying average linkage hierarchical clustering, we grouped genes exhibiting similar expression patterns into modules, ensuring a minimum gene group size of 100 based on the TOM- derived dissimilarity measure. In the final step, we amalgamated multiple modules after assessing the dissimilarity of their respective eigengenes and establishing a cut-off point within the module dendrogram. Through this process, we identified key module genes related to AD and created a visual representation of the eigengene network.

Through the processes described earlier, we obtained DEGs and identified key module genes related to the disease using WGCNA. By taking the intersection of these two sets of genes, we determined the key genes. The R package “venneuler” was utilized to generate a Venn diagram. These key genes were retained for further analysis.

### 2.5 Machine learning

To further identify the pivotal genes that are critical for the diagnosis of AD, this study used ten distinct machine-learning algorithms. Employing a 10-fold cross-validation methodology, these algorithms were randomly grouped together, resulting in the creation of a total of 113 combinational algorithms. The diagnostic ability of the pivot gene was validated using the ROC curve with GSE118553 as the training set and other datasets as the external validation sets. To identify the most effective combination, the average AUC for each signature was computed, allowing for the selection of the optimal algorithmic signature based on its performance metrics [27].

### 2.6 Immune infiltration analysis

In order to further explore the role of immune processes in the progression of AD, an immune infiltration analysis was performed in this study using a meta-data dataset. This analysis was based on the 7 algorithms using a reference set [28,29]. The results of the immune cell infiltration were visualized using the R language packages ‘ggplots’ and ‘pheatmap’.

### 2.7 eQTL datasets

Blood eQTL comprises a comprehensive collection of data reflecting the cis-eQTLs for a total of 16,987 genes. This dataset was generated from a substantial cohort of 31,684 blood samples. In the analysis, we focused on fully significant cis-eQTL results, which were established with a false discovery rate (FDR) of less than 0.05. Additionally, important allele frequency information was also gathered to support the findings [30]. Besides, brain eQTL specifically addressed samples from the prefrontal cortex, incorporating data from 1387 individuals, predominantly of European descent. We ensured that our analysis included all significant eQTLs with an FDR of less than 0.05, specifically targeting genes that displayed an expression level greater than 0.1 fragments per kilobase per million mapped fragments in at least 10 samples [31]. Furthermore, we collected all relevant SNP information to substantiate our research findings (Table 1).

**Table 1:** The details of markers associated GWASs used in the study.

Markers	Number of samples	Ancestry	PMID	GWAS Catalog ID
CSF Aβ	8074	European	36,066,633	GCST90129599
CSF p-tau	7798	European	36,066,633	GCST90129600
Circulating t-tau	14,721	European	35,396,452	GCST90095138
Total hippocampal volume	21,297	European	30,279,459	GCST006871
Right hippocampal volume	21,282	European	33,875,891	GCST90002641
Left hippocampal volume	21,282	European	33,875,891	GCST90002624
Cortical surface	33,992	European	32,193,296	GCST010282
Cortical thickness	33,992	European	32,193,296	GCST010281
White matter hyperintensity	21,381	European	33,875,891	GCST90003862
White matter integrity (fractional anisotropy)	17,663	European	32,358,547	GCST010102
White matter integrity (mean diffusivity)	17,467	European	32,358,547	GCST010103

### 2.8 AD GWAS dataset

Data on AD patients and their controls were obtained from the FinnGen database. Exposure data were gene expression and the outcome variable was AD [32]. r2<0.001, kb<10,000, and *P* < 5 × 10–8 were set to screen SNPs to avoid weak instrumental variable bias, and heterogeneity of IVs was tested using MR-egger [33]. A two-sample Mendelian randomization study was conducted using the “Two Sample MR” package to assess the causal role of genes in AD.

### 2.9 Markers associated with AD

To assess the potential involvement of the three genes associated with AD (*LIMK2, MAPK8*, and *NDUFV2*), which were identified through our MR analysis—we conducted an additional round of two-sample MR analysis. This analysis utilized consistent MR parameters to examine the eQTL for the three genes in both brain and blood tissues. In the subsequent phase of our analysis, we transitioned to evaluating the GWAS associated with the identified biomarkers as the exposure variable, while considering the GWAS data focusing on AD as the outcome variable [[34], [35], [36], [37]]. This step was designed to further understand the mediating effects of these biomarkers in the context of AD, thereby providing deeper insight into the underlying mechanisms that may contribute to the development of the disease.

### 2.10 Statistical analysis

Statistical analyses were performed using R software (version 4.3.0). Two-sample *t*-tests, paired samples *t*-tests, and Mann-Whitney tests were utilized to analyze differences between only two subgroups. A p-value less than 0.05 was considered statistically significant.

## 3 Results

### 3.1 Identification of molecular subtypes of AD

**Fig. 2:**
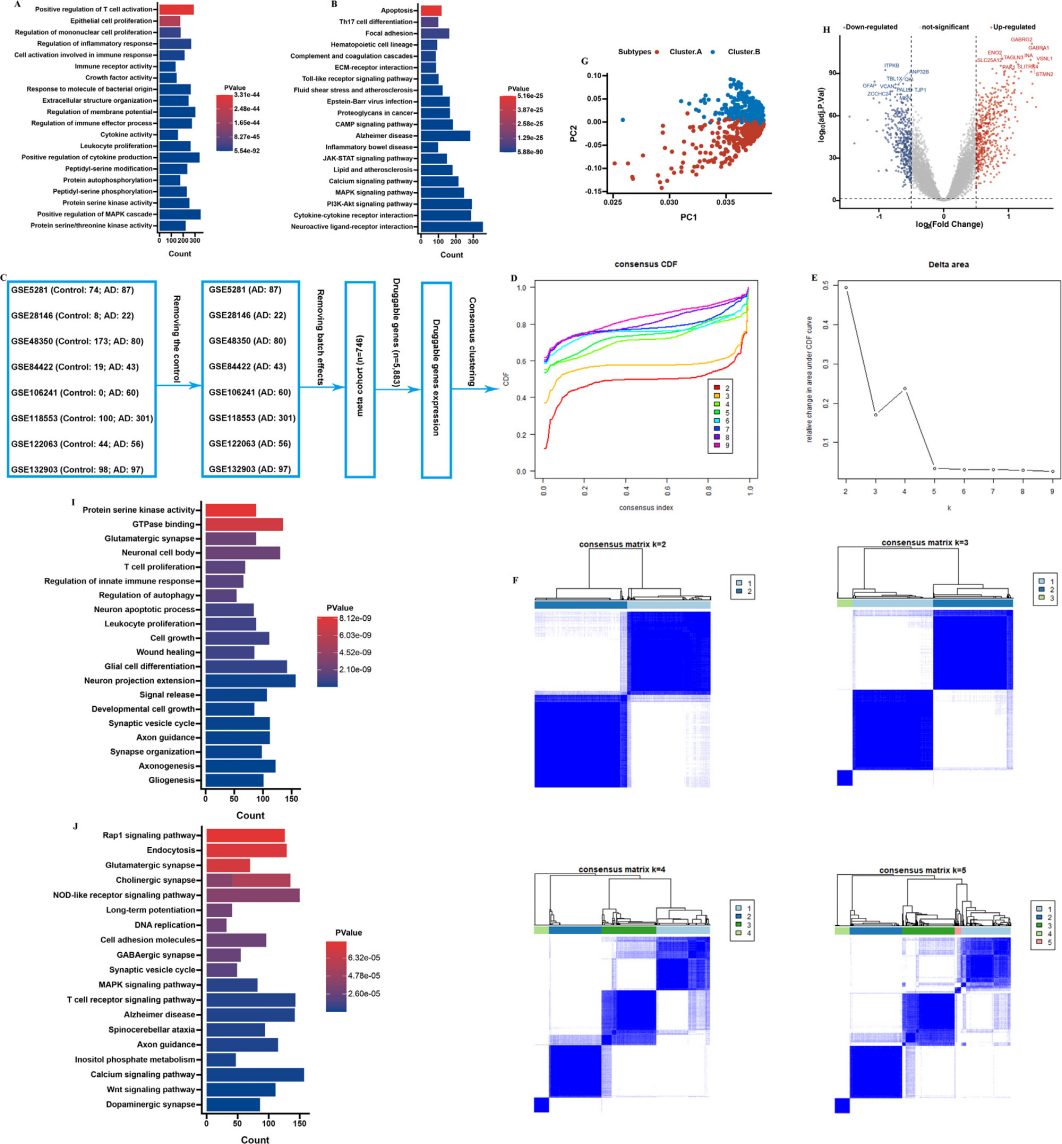
Identification of molecular subtypes of AD. (A) GO and (B) KEGG enrichment analysis on druggable genes. (C-E) Consensus clustering utilizing key druggable genes. CDF curve for *k* = 2–9 is shown. (F) The consensus score matrix of all samples when k = 2. (G) PCA analysis of difference between the 2 clusters. (H) DEGs between the 2 clusters. GO (I) and KEGG (J) enrichment analysis on the DEGs between the 2 clusters.

Firstly, in the analysis of druggable genes, GO (Gene Ontology) and KEGG (Kyoto Encyclopedia of Genes and Genomes) analysis shown that these genes were mostly involved in immune pathway (Fig. 2A and 2B). Then, we removed the controls from the 8 brain tissues datasets. Then, after removing batch effects, the datasets were merged into a meta cohort (Fig. 2C). Next, we utilized Consensus Cluster Plus package to identify distinct subgroups among 746 CE samples. The output revealed k (2 to 9) subgroups, successfully stratifying all patients into two subgroups based on the most stable k value. The results indicated that the best clustering occurred when *k* = 2 (Fig. 2D-F). To further investigate the potential biological functional impact between cluster.A (N = 353) and B (N = 393), we first performed DEGs analysis between clusters A and B and obtained 935 DEGs (Fig. 2G and 2H). GO and KEGG findings represented that these genes were involved in are AD enriched pathways and immune pathways, including glutamatergic synapse, neuronal cell body, neuron projection extension, and T cell proliferation (Fig. 2I and 2J).

### 3.2 Clinical of the AD subtypes

**Fig. 3:**
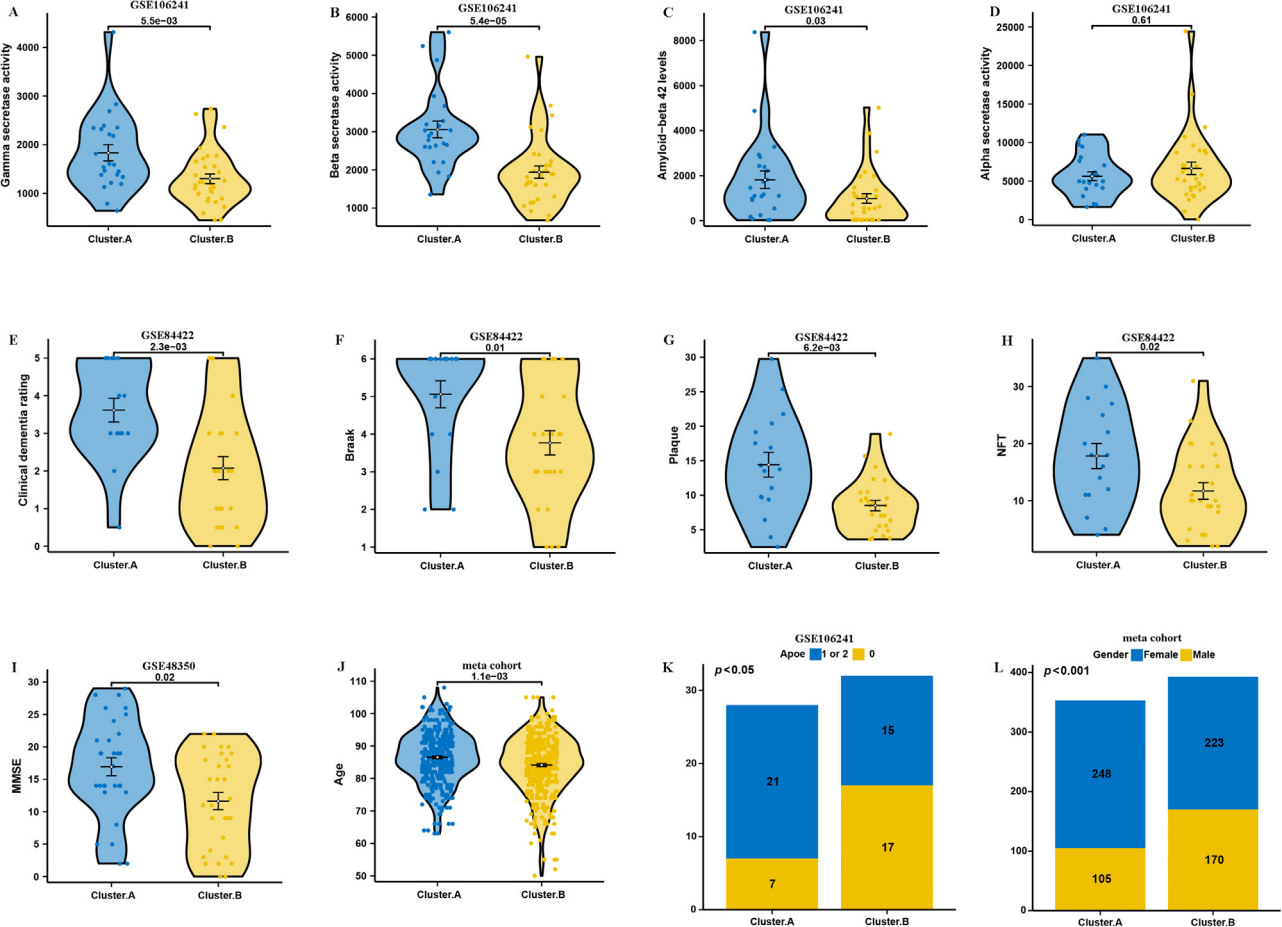
Clinical of the AD subtypes. Comparison of gamma-secretase activity (A), beta-secretase activity (B), amyloid−beta 42 levels (C), alpha secretase activity (D) between two AD subtypes in GSE106241. Comparison of Braak (E), plaque (F), NFT (G), clinical dementia rating (H), between two AD subtypes in GSE84422. Comparison of MMSE (I) between two AD subtypes in GSE48350. (J) Comparison of age between two AD subtypes in meta cohort. (K) Proportion of APOE between two AD subtypes in GSE106241. (L) Proportion of sex between two AD subtypes in meta cohort.

In our investigation, we conducted a comparative analysis of two subtypes of AD regarding their clinical characteristics. The levels of gamma secretase activity, beta secretase activity, and amyloid-beta 42 were found to be significantly elevated in patients classified within cluster A when compared to those in cluster B in GSE106241 dataset (Fig. 3A–D). These biochemical markers are critical in the pathology of Alzheimer's, suggesting a more pronounced disease progression in cluster.A patients. Moreover, findings from the GSE84422 dataset highlighted that patients in cluster.A experienced higher levels of Braak staging, plaque accumulation, neurofibrillary tangles (NFT), and clinical dementia rating compared to their counterparts in cluster.B (Fig. 3E–I). Such results indicate that cluster.A is associated with a more severe clinical presentation of dementia, pointing towards distinct pathological features linked to this subtype. Additionally, our meta-cohort analysis indicated that the age of patients in cluster.A was notably higher (Fig. 3J). This suggests that cluster.A patients may represent an older demographic, which could have implications for the progression and management of the disease. Furthermore, we observed a significant difference in genetic predisposition between the two clusters; specifically, the presence of APOE 4 alleles was markedly more prevalent in cluster.A within the GSE106241 dataset (Fig. 3K). The APOE 4 allele is a well-established genetic risk factor for AD, thus reinforcing the notion that cluster.A is characterized by biological and genetic factors that may contribute to its clinical severity. Lastly, the analysis revealed a significant disparity in gender distribution, with a higher proportion of female patients in cluster.A (Fig. 3L). This finding underscores the importance of considering sex as a variable in the study of AD, particularly in relation to its clinical manifestations and outcomes.

### 3.3 Immune cell infiltration between two molecular subtypes

**Fig. 4:**
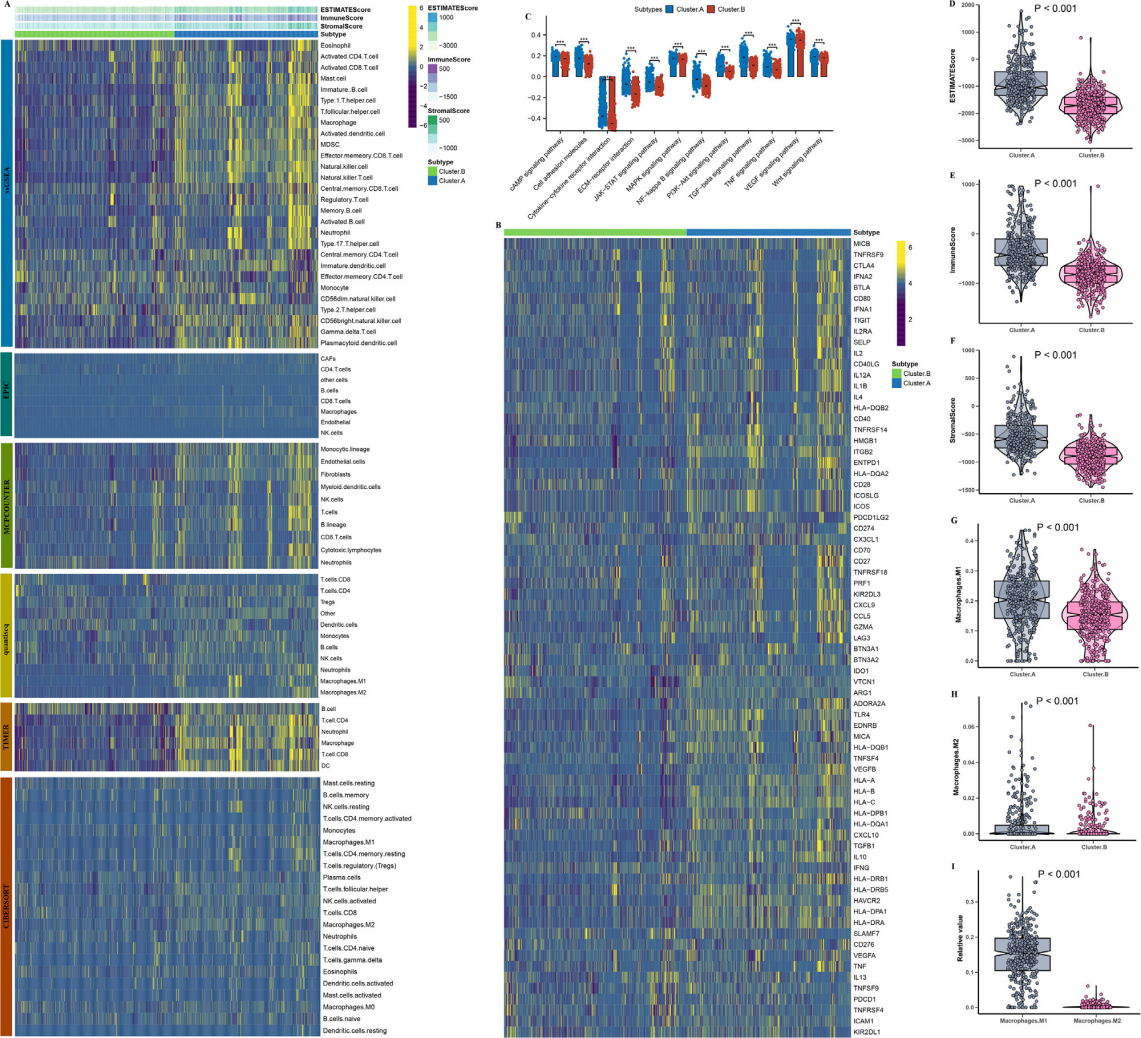
Immune cell infiltration between two molecular subtypes. (A) The immune landscape between the molecular subtypes. (B) The immune modulator molecules expression between the molecular subtypes. (C) Box plot displaying the main pathway activity between the two molecular subtypes. (D-I) Comparison of ImmuneScore, StromalScore, ESTIMATEScore, M1, and M2 between two molecular subtypes. (*P < 0.05; **P < 0.01; ***P < 0.001).

Next, by analyzing immune cell infiltration in cluster.A and cluster.B in order to explore potential links with host immune processes in AD. Then, we initially analyzed the overall stromal cell, immune cell infiltration levels of patients in the two molecular subtypes using the ESTIMATE algorithm, and immuneScore and stromalScore of cluster.A patients were higher (Fig. 4A–F). Subsequently, we analyzed the different between molecular subtypes various immune cell infiltrations. The results demonstrated cluster.A had higher immune infiltration abundance and immune check-points (Fig. 4A and 4B). Additionally, the analysis revealed that the activity levels of the primary signaling pathways were markedly greater in cluster.A (Fig. 4C). Besides, M2-like macrophages were markedly greater in cluster.B compared to cluster.A (Fig. 4G-I). These findings suggest that patients in the cluster.A category present an inflamed immune microenvironment in AD patients.

### 3.4 Identification of key module genes

**Fig. 5:**
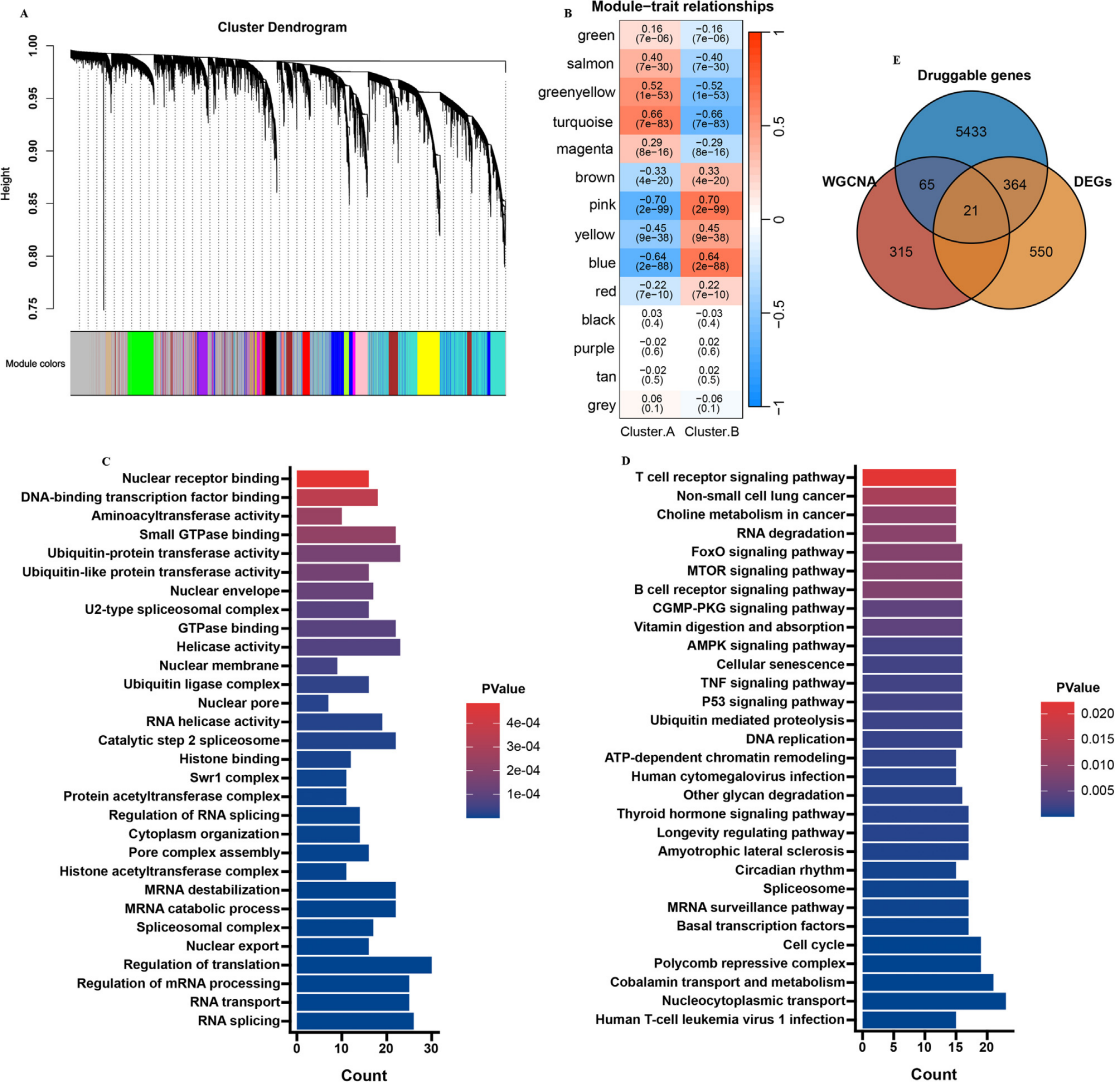
Identification of key module genes associated with molecular subtypes. (A) Identification of co-expression gene modules. (B) Heatmap showing the correlation between modules and feature gene sets. GO (C) and KEGG (D) enrichment analysis on the key module genes. (E) Venn diagram showing the markers intersected by cluster DEGs, key module genes, and druggable genes.

Next, WGCNA was employed to analyze the meta cohort, which comprises the largest number of samples. The present study constructed an adjacency matrix that followed a scale-free network by setting β=3 (R^2^=0.85) and maintaining high connectivity based on gene distribution. Then, 15 co-expression modules were screened (Fig. 5A). Heatmap revealed that the black module exhibited the most significant positive correlation with AD, and 401 genes were selected from the pink module (Fig. 5B). Then, GO and KEGG findings represented that these genes were involved in are enriched in DNA replication pathway (Fig. 5C and D).

### 3.5 Identification of hub genes by machine learning

**Fig. 6:**
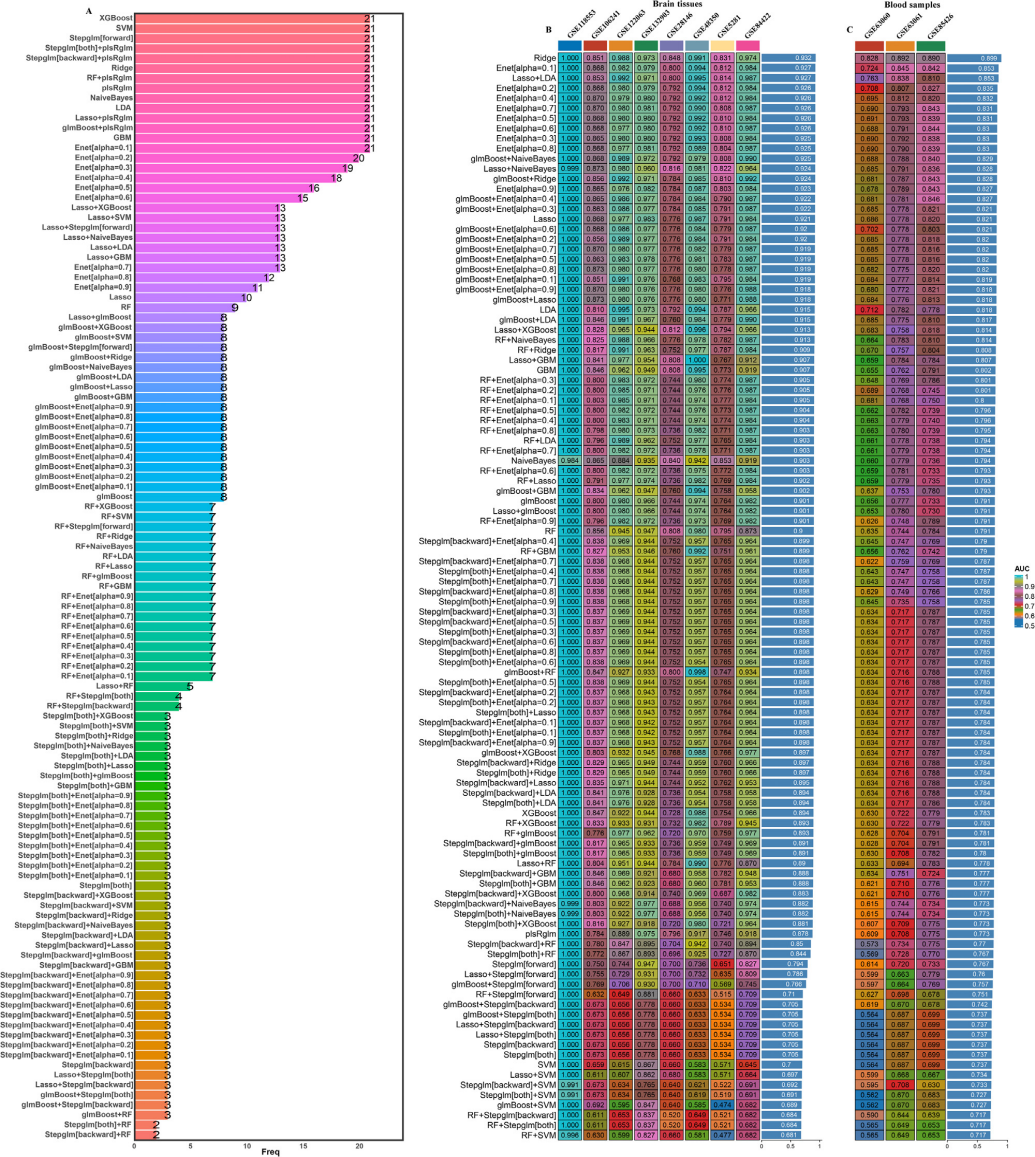
Identification of hub genes by machine learning. (A) The most valuable overlapping genes based on the multiple algorithms. (B) Hub genes were identified by a total of 113 combinations of machine learning algorithms.

Firstly, a gene set including 21 was obtained via overlapping DEGs between 2 subtypes, key module genes by WGCNA, and druggable genes (DG) for subsequent analysis (Fig. 5E). To delve deeper into the analysis of the 21 gene features, we implemented advanced artificial intelligence techniques, specifically employing ten distinct machine-learning algorithms alongside 113 different combinations. This rigorous process aimed to identify potential predictors associated with these gene features. Following this, we carefully calculated the expression levels of the 21 optimal hub genes, subsequently weighting them according to their regression coefficients. According to our methodology, we made an intriguing discovery: the Ridge model emerged as the most effective among all the artificial intelligence algorithms we tested, and 21 hub genes were identified (Fig. 6A). It achieved the highest average AUC value of 0.932 in AD brain tissues and 0.899 in AD blood samples (Fig. 6B and C).

### 3.6 Clinical characteristics of DG.score

**Fig. 7:**
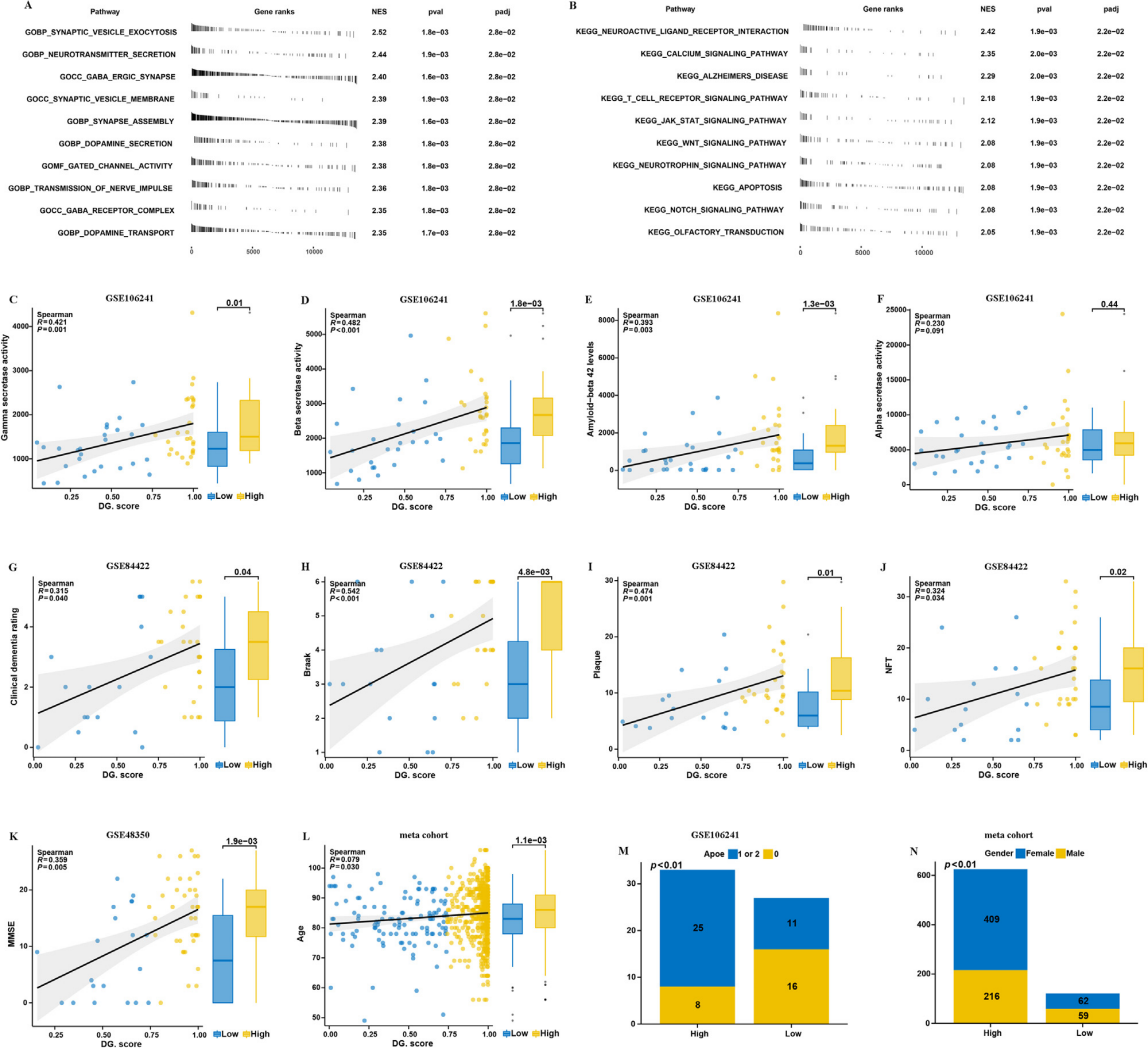
Clinical characteristics of DG.score (A) GSEA GO and GSEA KEGG enrichment analysis on high/low DG.score groups. (C—N) Analysis of differences of clinical characteristics between high/low DG.score groups.

Based on the mean value of DG.score, we categorize the sample into high DG.score group and low DG.score group. Utilizing GSEA, further analyses were conducted to explore the relevant pathways associated with high and low DG.score. High DG.score is primarily involved in synaptic vesicle exocytosis, neurotransmitter secretion, synapse assembly. These upregulated gene set likely reflect the activation of neuro pathways during the pathogenesis of AD (Fig. 7A and B). Next, we conducted a comparative analysis of high and low DG.score regarding their clinical characteristics. The data derived from the GSE106241 dataset revealed that high DG.score exhibited increased levels of gamma secretase activity, beta secretase activity, and amyloid-beta 42 when compared to those in low DG.score (Fig. 7C–F). Moreover, findings from the GSE84422 dataset highlighted that patients in high DG.score experienced higher levels of Braak staging, plaque accumulation, NFT, and clinical dementia rating compared to their counterparts in low DG.score (Fig. 7G–K). Additionally, meta-cohort indicated that the age of high DG.score was notably higher (Fig. 7L). Furthermore, we observed a significant difference in genetic predisposition between the two groups; specifically, the presence of APOE 4 alleles was markedly more prevalent in high DG.score within the GSE106241 dataset (Fig. 7M). Lastly, the analysis revealed a significant disparity in gender distribution, with a higher proportion of female patients in high DG.score (Fig. 7N).

### 3.7 MR analysis

**Fig. 8:**
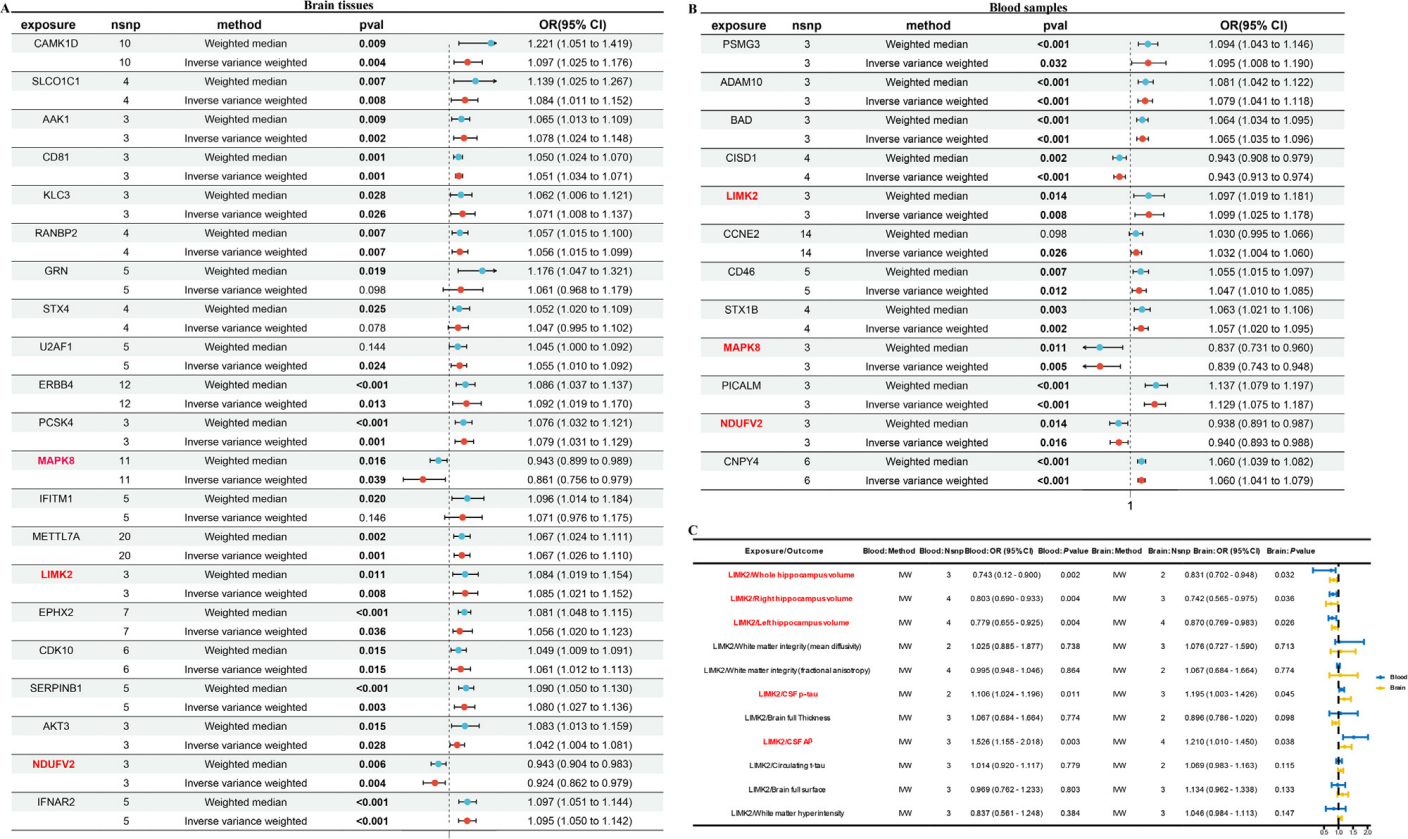
Identification of hub genes in AD by MR. (A) Forest plot for MR results between brain eQTL and AD. (B) Forest plot for the MR result between blood eQTL and AD. (C) MR results of AD markers and AD outcome in IVW method.

Next, *LIMK2, MAPK8*, and *NDUFV2* might contribute to an enhanced risk of AD by MR in brain tissues and blood samples. They all showed risk influence on AD (*LIMK2* OR 1.084 (1.019–1.154), *MAPK8* OR 0.943 (0.899–0.989), and *NDUFV2* OR 0.943 (0.904–0.983)) in brain tissues (Fig. 8A), and (*LIMK2* OR 1.097 (1.019–1.181), *MAPK8* OR 0.837 (0.731–0.960), and *NDUFV2* OR 0.938 (0.891–0.987)) in blood samples (Fig. 8B). Our research indicates a potential correlation between the levels of *LIMK2* and overall volume of the hippocampus (Fig. 8C). This relationship suggests that *LIMK2* could play a significant role in reducing hippocampal size (OR 0.831 (0.702–0.948)), which is an important factor in various cognitive functions and neurological health. Further investigation into this association may enhance our understanding of the underlying mechanisms connecting *LIMK2* to hippocampal development and function. Furthermore, our research also revealed a potential association between the levels of *LIMK2* and the concentrations of amyloid-beta (Aβ) (OR 1.526 (1.155–2.018)) and phosphorylated tau protein (p-tau) (OR 1.106 (1.024–01.196)) in cerebrospinal fluid (CSF). This finding suggests that *LIMK2* could play a significant role in the biological mechanisms underlying these important biomarkers often linked to neurodegenerative conditions, particularly AD.

## 4 Discussion

AD is a chronic neurodegenerative disorder, and its incidence has been steadily increasing alongside the intensifying global aging population [38]. AD places considerable financial strain on both patients and their families, and also represents a significant obstacle for healthcare systems across the globe. Nevertheless, the quest to develop new therapeutic agents for AD presents significant challenges that are difficult to overcome. One of the primary reasons for this complexity is the insufficient understanding of the disease's underlying pathophysiology. Without a clear grasp of how AD progresses and the biological mechanisms at play, researchers face considerable obstacles in identifying effective treatments. This uncertainty complicates the research and development process, making it difficult to target the disease effectively and find appropriate interventions. Therefore, the development of novel drugs for the treatment of AD is extremely challenging.

In this study, we conducted a comprehensive bioinformatics analysis of microarray datasets from AD patients and GWAS data to identify potential therapeutic target for AD. In our investigation, we identified three druggable genes, *LIMK2, MAPK8*, and *NDUFV2*, associated with expressions that may have an impact on the outcomes of AD. Furthermore, our research also revealed a potential association between the levels of *LIMK2* and the concentrations of CSF Aβ, CSF p-tau, and hippocampal size. This discovery is particularly noteworthy as it adds a valuable dimension to the existing body of literature. By presenting genetic evidence that underscores the potential therapeutic advantages of focusing on these specific druggable genes, our research highlights a promising avenue for the treatment of AD. This insight will be instrumental in guiding future efforts in drug development aimed at addressing this AD.

Utilizing consensus unsupervised clustering analysis, we identified two AD subclasses and intersected them with DEGs to derive AD-related key genes. Subsequent GO and KEGG analyses of these genes revealed strong associations with AD pathogenesis, highlighting immune responses, neuronal microtubule maintenance, glucose metabolism abnormalities, and related cell signaling pathways [39,40]. Meanwhile, the proportions of immune cells in clusters.A patients were significantly higher than those of clusters.B patients. Recent findings highlight the significant impact of immune dysregulation, especially the infiltration of immune cells, in the development of AD. The abnormal accumulation of immune cells in the brains of AD patients has emerged as a pivotal factor in disease progression [41]. While these cells aid in clearing harmful substances like amyloid-β plaques and neurofibrillary tangles, they can paradoxically promote neuronal damage through the release of proinflammatory factors and other pathways. When this immune response becomes deregulated, it can lead to chronic inflammation and further neuronal damage [42] Therefore, a profound understanding of the immunological mechanisms underlying AD, particularly the role of immunological infiltration in disease progression, is imperative for developing novel therapeutic strategies.

Utilizing WGCNA, we identified clinically relevant modules and intersected them with DEGs to derive AD-related key genes. Specifically, the KEGG analysis revealed potential associations between AD-related key genes and pathways involved in neuroinflammation. Astrocytes secrete various immunomodulatory molecules, including cytokines and chemokines, which play vital roles in inflammatory responses and neurodegeneration, regulating the immune system's reactivity. Previous findings have implicated the involvement of immune responses in AD, aligning with the growing literature highlighting the intricate link between the immune system and multiple neurological disorders. Abnormal activation of neuroimmune cells and heightened inflammatory reactions significantly contribute to the progression of AD.

Through machine learning approaches, a diagnostic model was constructed and 21 key genes were screened out, and these 21 key genes were able to distinguish AD tissues from healthy control tissues in both the training and validation sets. Specifically, ROC analysis revealed DG.score's high diagnostic value for AD, with AUC values exceeding 0.7 in all brain tissues datasets and blood samples datasets, indicating its high sensitivity and specificity (brain tissues datasets: GSE118553: AUC = 1.000; GSE106241: AUC = 0.851; GSE122063: AUC = 0.988; GSE132903: AUC = 0.973; GSE28146: AUC = 0.848; GSE48350: AUC = 0.991; GSE5281: AUC = 0.831; GSE84422: AUC = 0.974. blood samples datasets: GSE63060: AUC = 0.828; GSE63061: AUC = 0.892; GSE85426: AUC = 0.890). Subsequently, LIMK2 was associated with the development of AD based on MR.

LIM kinases (LIMKs) are a class of serine/threonine kinases that include two members, *LIMK1* and *LIMK2*. The protein structure consists of two LIM domains, a PDZ domain, and a kinase domain [43]. Studies have shown that LIMKs can participate in the regulation of cell activity and function by regulating the cell skeleton, transcriptional regulatory factors. On the one hand, it can regulate the function of the cytoskeleton, affecting the migration and invasion of cells [44]. On the other hand, within the nervous system, it can affect the structure and function of neurons by regulating the expression of cofilin and the of cyclic *AMP* response element-binding protein (*CREB*), thereby interfering with synaptic transmission and changes in synaptic plasticity [45]. Since LIMK is a common pathway regulated by the G-protein family to regulate the actin cytoskeleton, LIMK becomes a therapeutic target for related diseases. *LIMK1* plays a role in the processes of tumor cell invasion and migration, and its phosphorylation can cause changes in the dynamics of act in the cytoskeleton and depolymerization of microtubules [46]. *LIMK2* may be involved in cytoskeletal remodeling, it can promote the synthesis of actin, and phosphorylate cofilin to it from depolymerizing filamentous actin (F-actin) [47]. *LIMK2* is located downstream of the Rho-ROCK signaling pathway and its main function is to regulate the reorganization of act filaments and plasma membrane structures by phosphorylating cofilin and ADF proteins. *LIMK2* is co-activated by both active *Rho* and *CDC42*, which induce the formation of stress fibers and filopodia, respectively. Therefore, activation of the Rho-ROCK-LIMK2-cofil signaling pathway can lead to changes in the actin cytoskeleton [48]. The active form of LIMK, p-LIMK, appeared at the axonal tips, the region where obvious morphological changes occurred injury. Inspection of brains from patients with AD revealed an increase in p-LIMK-positive neurons in the AD-affected areas. Treatment hippocampal neurons with fibrillar amyloid-β induced actin filament reorganization, dystrophy, and cell death. This cellular p-LIMK and p-cofilin expression was increased, while inhibition of LIMK-mediated cofilin phosphorylation blocked actin reorganization and neuronal dystrophy induced byillar amyloid-β treatment [49]. This further highlights the importance of *LIMK2* in the nervous system, thus providing new insights for clinical diagnosis and treatment.

However, our study has certain limitations that warrant further discussion. Firstly, while we have not validated our findings at the molecular level, functional validation in animal models and clinical trials is necessary to fully elucidate the role of *LIMK2* in AD pathogenesis. Secondly, the immune cell infiltration analysis was based on bioinformatics algorithms and requires further confirmation using immunological techniques. Finally, the complexity of AD pathogenesis necessitates a more comprehensive analysis of the interactions between *LIMK2* and other genes/proteins involved in the disease.

In conclusion, we performed a comprehensive investigation into the potential drug targets among druggable genes related to AD by employing integrated bioinformatics analyses. This research offers genetic insights that endorse the possible therapeutic advantages of focusing on *LIMK2* in the treatment of AD. Additionally, the findings highlighted variations in the patterns of immune cell infiltration and the differences in enriched pathways among various AD subtypes. Therefore, an in-depth study of *LIMK2* in AD contributes to a deeper comprehension of AD pathogenesis and proposes effective therapy strategies.

## Ethics approval and consent to participate

Not applicable.

## Consent for publication

Not applicable.

## Funding

This work was received no funding.

## Authors’ contributions

JCH and XCW designed the study, performed statistical analysis, and drafted the manuscript. YL helped to draft the manuscript. All authors read and approved the final manuscript.

## Declaration of generative AI and AI-assisted technologies in the writing process

I confirm that I have not used any AI at all.

## CRediT authorship contribution statement

**Jichang Hu:** Writing – review & editing, Writing – original draft, Project administration. **Yong Luo:** Validation, Software, Methodology, Formal analysis, Data curation. **Xiaochuan Wang:** Validation, Software, Methodology, Formal analysis.

## Declaration of competing interest

The authors declare that they have no known competing financial interests or personal relationships that could have appeared to influence the work reported in this paper.
